# IL-24 producing regulatory T and B lymphocytes in endometriosis

**DOI:** 10.3389/fimmu.2025.1582762

**Published:** 2025-06-18

**Authors:** Anna Ewa Kedzierska, Daria Lorek, Anna Slawek, Mikolaj Karmowski, Aleksandra Kalota, Jaroslaw Pajak, Agnieszka Chrobak, Krzysztof Grzymajlo, Anna Chelmonska-Soyta

**Affiliations:** ^1^ Department of Experimental Therapy, Hirszfeld Institute of Immunology and Experimental Therapy, Polish Academy of Sciences, Wrocław, Poland; ^2^ Department of Immunology, Pathophysiology and Veterinary Preventive Medicine, Faculty of Veterinary Medicine, Wrocław University of Environmental and Life Sciences, Wrocław, Poland; ^3^ Endometriosis Institute of EuroMediCare Specialist Hospital, Wrocław, Poland; ^4^ 1st Department of Gynecology and Obstetrics, Wrocław Medical University, Wrocław, Poland; ^5^ Clinical Department of Oncological and Procreative Gynecology of the 4th Military Clinical Hospital with the Polyclinic, Wrocław, Poland; ^6^ Medical Faculty, Wrocław University of Science and Technology, Wrocław, Poland; ^7^ Department of Biochemistry and Molecular Biology, Faculty of Veterinary Medicine, Wrocław University of Environmental and Life Sciences, Wrocław, Poland

**Keywords:** IL-24, Bregs, Tregs, endometriosis, plasmablasts, B10, immature B cells

## Abstract

**Problem:**

Unbalanced production of pro- and anti-inflammatory cytokines by immune cells is a hallmark of endometriosis. IL-24, a member of the IL-10 family, is a pleiotropic cytokine produced by both non-immune cells like astrocytes, keratinocytes, pancreatic myofibroblasts, and endothelial cells and immune cells such as monocytes, macrophages, dendritic cells, NK cells, T cells (including Th2 and Th17), and B cells. However, its expression in regulatory T (Tregs) and B lymphocytes (Bregs) has not been explored. In this study, we determined the expression of IL-24 in Tregs and selected Breg subpopulations in women with endometriosis compared with healthy women.

**Methods:**

Percentages of Tregs, B10 cells, immature B cells, and plasmablasts that produce IL-24 were measured in the peripheral blood of women with endometriosis (n=24) and healthy women (n=24) using flow cytometry.

**Results:**

We observed an increased percentage of IL-24–producing Tregs in the total pool of women with endometriosis and in women with stages III and IV of endometriosis compared to controls. Within the Breg subpopulations, the percentages of IL-24–producing plasmablasts were higher in the overall endometriosis cohort as well as in women with stage IV endometriosis compared with healthy women. In contrast, the percentages of IL-24–producing immature B cells were lower in the endometriosis group than that in the control group.

**Conclusions:**

We have shown, for the first time, that Tregs and Bregs secrete IL-24 and that their percentages are altered in endometriosis. The significance of this cytokine secretion by regulatory cells is unclear, but we speculated that IL-24 may enhance the improper immunosuppressive activity of Tregs and plasmablasts in endometriosis, which enables the implantation and growth of endometrial lesions outside the uterus.

## 1 Introduction

Endometriosis is a chronic disease characterized by the presence of uterine tissue outside the uterine cavity and persistent inflammation. Endometriosis is accompanied by chronic pelvic pain, dyspareunia, dysmenorrhea, and infertility (1–3). This condition affects up to 10% of the female population and as many as 50% of infertile women (4–9). The pathogenesis of endometriosis remains ambiguous; nevertheless, the most widely accepted hypothesis is retrograde menstruation, which implies that during menstruation, the endometrial lining migrates through the fallopian tubes into the pelvic space (10). These endometrial fragments may implant and survive at ectopic sites. However, retrograde menstrual blood is relatively common in women while endometriosis affects only a few women, suggesting that other factors such as genetic, hormonal, environmental, or immunologic are involved in the implantation and survival of endometrial lesions in the peritoneal cavity.

Abnormal immune responses, particularly the dysfunction of peripheral and peritoneal cavity immune cells that secrete various cytokines, chemokines, and growth factors, have been associated with the development of endometriosis (11–14). Therefore, women suffering from endometriosis exhibit chronic systemic inflammation, which is manifested by altered pro-inflammatory cytokines (IL-1β, IL-6, IL-8, and TNF-α), T cell cytokines (IL-12, IL-17, and IFN-γ), and anti-inflammatory cytokines like IL-4 and IL-10 (15–24). Among these, members of the IL-10 family have been implicated in suppressing immune response against endometriotic fragments, thereby contributing to the development of endometriosis (24). Regulatory T (Tregs) and B cells (Bregs), which secrete IL-10 and establish self-tolerance, play a key role in this process. The percentage of Tregs is significantly higher in the peritoneal fluid of women with endometriosis compared with healthy women (25, 26) and increases with disease progression (27). Moreover, elevated percentages of circulating Tregs have been found in patients with endometriosis than in controls (28). Recently Le et al., 2021 have shown no difference in peripheral natural Tregs and inducible Tregs between patients with endometriosis and controls (29). Furthermore, other findings indicate no significant variation in circulating Tregs during the menstrual cycle between women with and without endometriosis (30). The discrepancies between studies may be due to the low number of cases, the use of heterogeneous methodologies, and the fact that Tregs themselves constitute a heterogeneous group of T cells (31). In contrast, the percentages of Breg subpopulations at different developmental stages are lower in women with endometriosis than in healthy women (32).

Recently, altered levels of interleukin-24 (IL-24), a member of the IL-10 cytokine family also known as melanoma differentiation-associated gene-7 (mda-7), have been determined in the endometrium of women with endometriosis (33). IL-24 primarily functions as a tumor-suppressive cytokine via multiple mechanisms, including inhibition of invasion, migration, angiogenesis, and metastasis, induction of apoptosis, elimination of cancer stem cells, and sensitization of cancer cells to therapies (34–40). However, the physiological role of IL-24 is still being investigated. Various immune cells, such as monocytes, macrophages, dendritic cells, NK cells, T cells (including Th2 and Th17), and B cells, can express IL-24 upon stimulation with cytokines, concanavalin A, lipopolysaccharide, 12-myristate 13-acetate (PMA), and/or PMA/ionomycin (41–46). However, no data has focused on the ability of regulatory T and B lymphocytes to secrete IL-24. Therefore, we investigated whether Tregs and several subpopulations of Bregs at different developmental stages can produce IL-24 upon PMA/ionomycin stimulation and whether their percentages are altered in patients with endometriosis.

## 2 Methods

### 2.1 Patients

This study was approved by the Bioethical Commission of the Medical University of Wrocław (No. KB – 407/2018), and written informed consent was obtained from all participants to participate and process personal data for research purposes. Peripheral venous blood from healthy women (n=24) were obtained from the Hirszfeld Institute of Immunology and Experimental Therapy, Polish Academy of Sciences. Blood samples from women with endometriosis (n=24) were collected from MultiMedica Clinics, Wrocław, and Clinical Department of Oncological and Procreative Gynecology, 4th Military Clinical Hospital in Wrocław. Women in the endometriosis group (aged 23–38 years old) were scheduled for laparoscopic surgery for suspected endometriosis based on specific pain symptoms and/or infertility evaluation. Women in the control group (aged 23–38 years old) had no history of endometriosis, pain symptoms, or pelvic abnormalities. Exclusion criteria included the presence of neoplastic disease, autoimmune disease, and pregnancy. A total of 48 women were enrolled in this study (**Table 1**). No differences were found in patients’ age, body mass index (BMI), menstrual cycle phases, or hormonal therapy for hypothyroidism (p > 0.05) between the examined groups. Endometriosis stage was assessed according to the ASRM classification (47). Samples were exclusively collected from women in advanced stages of endometriosis (stages III and IV), as collecting samples from patients in early stages of the disease (stages I and II) was challenging because of the typical diagnostic delay associated with the disease.

**Table 1:** Characteristics of patients enrolled in the study.

Patient characteristics	Control N=24 (% (n/N))	Endometriosis N=24 (% (n/N))	Stage III N=8 (% (n/N))	Stage IV N=16 (%(n/N))
**Woman’s age (mean ± SD)**	**28.8 ± 4.2**	**30.5 ± 4.6**	**32.1 ± 5.3**	**29.7 ± 4.0**
20–29	70.8% (17/24)	41.7% (10/24)	22.2% (2/8)	50% (8/16)
30–34	16.7% (4/24)	33.3% (8/24)	44.5% (4/8)	31.2% (5/16)
35–40	12.5% (3/24)	25% (6/24)	33.3% (3/8)	18.8% (3/16)
**Women’s BMI, kg/m^2^ (mean ± SD)**	**21.41± 2.3**	**21.2 ± 2.2**	**21.9 ± 2.3**	**20.6 ± 2.0**
underweight (<18.5)	20.8% (5/24)	8.3% (2/24)	0.0% (0/8)	11.1% (2/18)
normal weight (18.5–24.9)	75% (18/24)	83.3% (20/24)	75% (6/8)	88.9% (16/18)
overweight (25–29.9)	4.2% (1/24)	8.3% (2/24)	25% (2/8)	0.0% (0/18)
**Hypothyroidism**	12.5% (3/24)	8.3% (2/24)	0% (0/8)	12.5% (2/16)
**Hormonal therapy**	8.3% (2/24)	16.7 (4/24)	25% (2/8)	12.5% (2/16)
**Follicular phase of menstrual cycle**	50% (12/24)	58.3% (14/24)	50% (4/8)	62.5% (10/16)
**Luteal phase of menstrual cycle**	50% (12/24)	41.7% (10/24)	50% (4/8)	37.5% (6/16)

N, group size; n, number of patients in the subgroup; SD, standard deviation. Data are expressed as mean ± standard deviation. Normality was assessed using Shapiro–Wilk normality test. p > 0.05 as analyzed by Student’s t-test or One-way ANOVA for BMI and age; p > 0.05 as analyzed by or chi2 test. p > 0.05 for menstrual cycle phases, hormonal therapy, hypothyroidism.Parameter under study is indicated in bold.

### 2.2 Flow cytometry

Blood samples were collected into lithium heparin collection tubes and cultured in Roswell Park Memorial Institute (RPMI) 1640 medium (Biowest, France) supplemented with 0.1 μg/ml phorbol 12-myristate 13-acetate (Cayman, USA), 1 μg/ml ionomycin (Cayman, USA), 10 μg/ml Brefeldin A (Biolegend, USA), and 2 μM Monensin A (Biolegend, USA) for 4 h at 37°C in 5% CO_2_. After stimulation, samples were stained with Zombie Red Viability Dye (Biolegend, USA) for 15 min at 4°C in the dark. Anti-human CD19 FITC (Biolegend, clone: HIB19), CD24 APC-Cy7 (Biolegend, clone: ML5), CD27 Pacific Blue (Biolegend, clone: M-T271), and CD38 Alexa Fluor 700 (Biolegend, clone: HB-7) antibodies were added for Bregs phenotyping, whereas anti-human CD4 Pacific Blue (Biolegend, clone: OKT4), CD25 APC (Invitrogen, clone: CD25-3G10), and CD127 Alexa Fluor 700 (Biolegend, clone: A019D5) antibodies were used for Tregs phenotyping. Samples were then washed with staining buffer (0.5 mM EDTA, 0.002% sodium azide, 1% fetal bovine serum) and fixed with a fixation buffer (eBioscience, USA) for 14 h at 4°C in the dark. After two washes with permeabilization buffer (eBioscience, USA), samples were blocked with Human True Stain FcX (Biolegend, USA) for 10 min at 4°C in the dark. Samples for Bregs phenotyping were stained with anti-IL-10 APC (Biolegend, clone: JES3-19F1) and anti-IL-24 Biotin (BAF1965; R&D, USA) antibodies and those for Tregs phenotyping were stained with anti-Foxp3 PE-Cy7 (Invitrogen, USA; clone PCH101) and anti-IL-24 Biotin (BAF1965; R&D, USA) antibodies or normal goat IgG biotinylated control antibody (BAF108, R&D, USA) for 1 h at 4°C in the dark. Samples were washed twice with permeabilization buffer and stained with Streptavidin PE (Biolegend, USA) for 30 min at 4°C in the dark. After washing again with permeabilization buffer, samples were analyzed using an LSR Fortessa Cell Analyzer (Becton Dickinson, USA). A total of 200,000 events were recorded at a rate of 600–800 events per second. Cytometer Setup and Tracking beads (CS&T Research Beads, Becton Dickinson, USA) were used for automated quality assurance and control of machine performance. Data were analyzed using FlowJo™ software version 10.8.1 (Becton Dickinson, USA). The relative levels of IL-24 in CD4^+^CD127^-^CD25^hi^FOXP3^+^ cells, CD19^+^CD27^+^CD24^hi^ cells, CD19^+^CD38^hi^24^hi^ cells, and CD19^-^CD38^hi^27^int^ cells were calculated as follows: median fluorescence of cells stained with anti-IL-24 antibody minus median fluorescence of cells stained with isotype-matched control. FlowJo tSNE plugin software was used for the two-dimensional reduction of the manual gates. Classical gating and tSNE algorithms (48) were used to assess IL-24-positive subpopulations. The percentage of each analyzed subpopulation producing IL-24 was calculated as the total number of viable cells, CD4^+^ or CD19^+^ cells or the parent population during gating. For 2-dimensional reduction, data from each patient were evenly downsampled and concatenated into a new data file, and then the tSNE algorithm was applied with the following settings: perplexity - 200; learning rate - 32200; iterations - 1000; and theta - 0.5.

### 2.3 Statistical analysis

All statistical analyses were performed using the GraphPad Prism 7 software (GraphPad Software, USA). Normal distribution was assessed using the Shapiro–Wilk normality test, while homoscedasticity was examined using the Brown-Forsythe test. Normally distributed data were examined using Student’s *t*-test (control group vs. endometriosis group) or one-way ANOVA (control group vs. stage III vs. stage IV endometriosis) (parametric). Non-normally distributed data were analyzed using Mann-Whitney *U* or Kruskal–Wallis test (non-parametric). One-way ANOVA and Kruskal–Wallis tests were followed by Tukey’s and Dunnett’s multiple comparisons *post-hoc* tests, respectively. The distribution of women in the menstrual cycle phases, receiving hormonal therapy for hypothyroidism, or receiving hormonal contraception was tested using chi^2^ test. Differences were considered statistically significant at p < 0.05. The statistical power was calculated based on the mean values of IL-24-producing Tregs percentages and assuming a significance level of 0.05 using the online calculator available at https://clincalc.com/stats/Power.aspx. The calculated power was 78.3%.

## 3 Results

### 3.1 IL-24 is produced by different Breg subpopulations and women with endometriosis have higher percentages of IL-24^+^ plasmablasts

**Figure 1:**
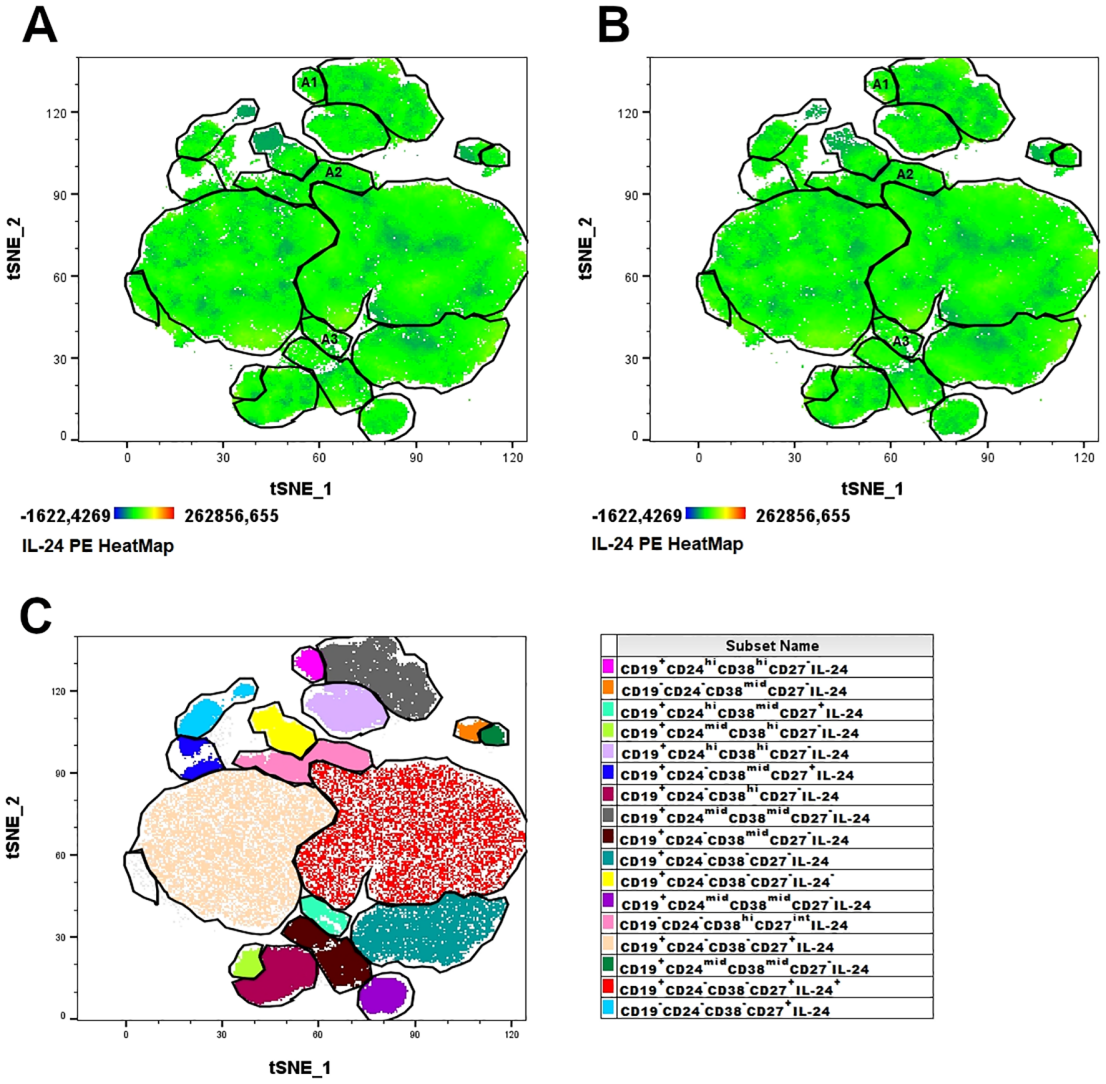
Combined t-SNE heatmap of IL-24–producing B cells. Combined t-SNE analysis was performed using 24 paired samples obtained from healthy women **(A)** and women with endometriosis **(B)**. **(C)** t-SNE plot presenting 17 distinct clusters of IL-24–producing cells identified using FLOWSOM algorithm. Regulatory B lymphocytes (Bregs) clusters were identified in A1 (immature B cells, CD19^+^CD24^hi^CD38^hi^CD27^-^IL-24^+^), A2 (plasmablasts, CD19^-^CD38^hi^CD27^int^CD24^-^IL-24^+^) and A3 (B10 cells, CD19^+^CD38^mid^CD24^hi^CD27^+^IL-24^+^) clusters.

We investigated specific subpopulations of B lymphocytes, namely Bregs, to determine their ability to produce IL-24 and whether their percentages varied in women suffering from endometriosis. First, we visualized the population of IL-24–producing B cells in healthy controls (**Figure 1A**) and women with endometriosis (**Figure 1B**) using t-distributed stochastic neighbor embedding (tSNE) algorithms. Next, the FLOWSOM algorithm was used to identify distinct clusters of IL-24–producing cells (**Figure 1C**). Heatmap of IL-24 expression revealed that distinct Breg subpopulations were located in specific clusters: A1 (CD19^+^CD24^hi^CD38^hi^CD27^-^IL-24^+^), A2 (CD19^-^CD24^-^CD38^hi^CD27^int^IL-24^+^), and A3 (CD19^+^CD24^hi^CD38^mid^CD27^+^IL-24^+^). tSNE analysis, presented as IL-24 heatmaps of different B cell subsets, revealed IL-24 expression in all analyzed clusters and similarity in complexity and distribution of IL-24 expression in healthy controls (**Figure 1A**) and women with endometriosis (**Figure 1B**).

**Figure 2:**
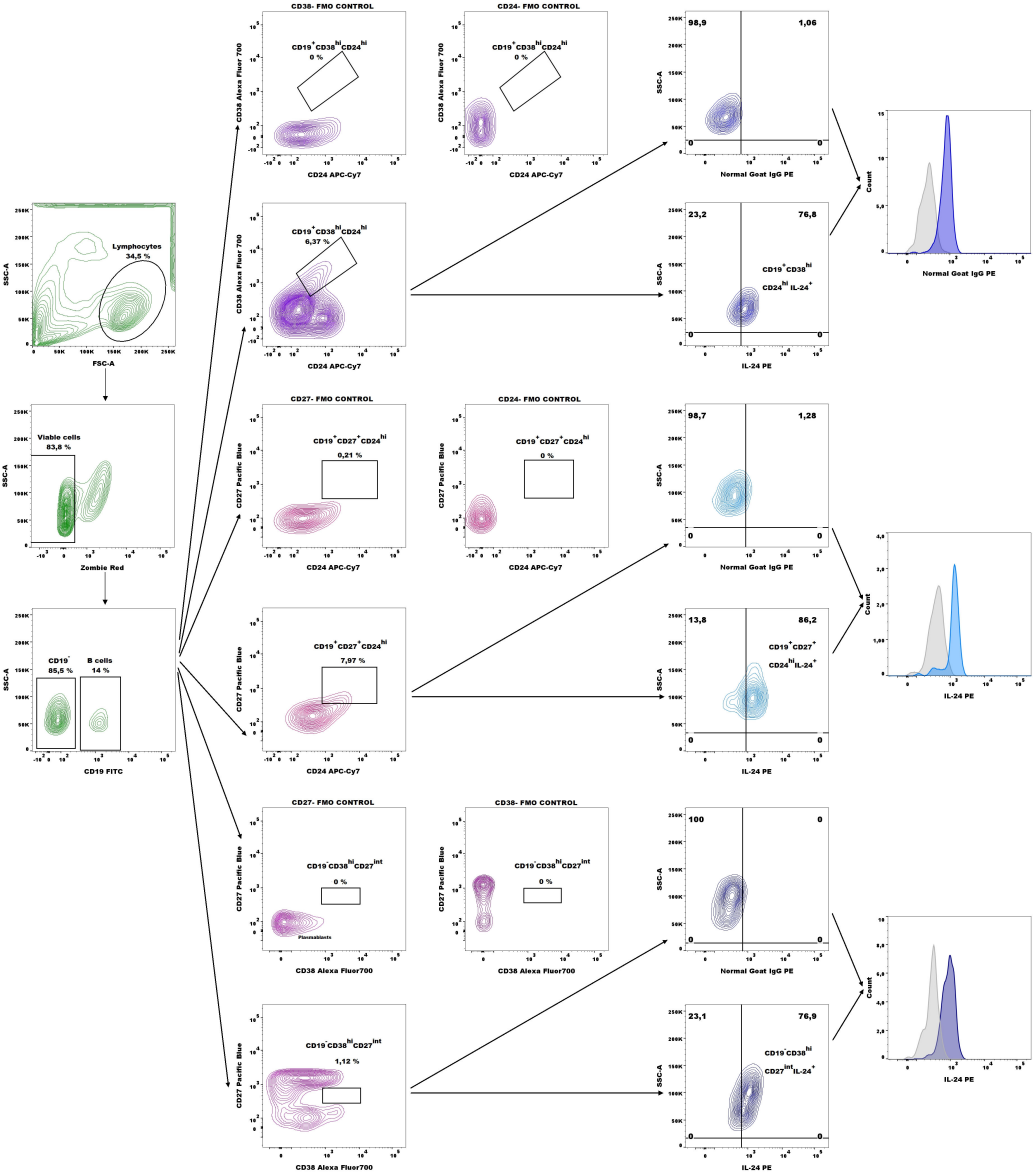
Representative dot plots for IL-24–expressing B10 cell (CD19^+^CD27^+^CD24^hi^), immature B cells (CD19^+^CD38^hi^CD24^hi^), and plasmablasts (CD19^-^CD38^hi^CD27^int^) gating and representative histograms of IL-24 expression (blue histograms) overlaid with those in the respective isotype-matched controls (grey histograms) derived from whole blood of patients with endometriosis.

Next, the number of CD19^+^CD27^+^CD24^hi^IL-24^+^ (B10 cells), CD19^+^CD38^hi^CD24^hi^IL-24^+^ (immature B cells), and CD19^-^CD38^hi^CD27^int^IL-24^+^ (plasmablasts) cells in whole peripheral blood were analyzed based on the gating strategy shown in **Figure 2**.

**Figure 3:**
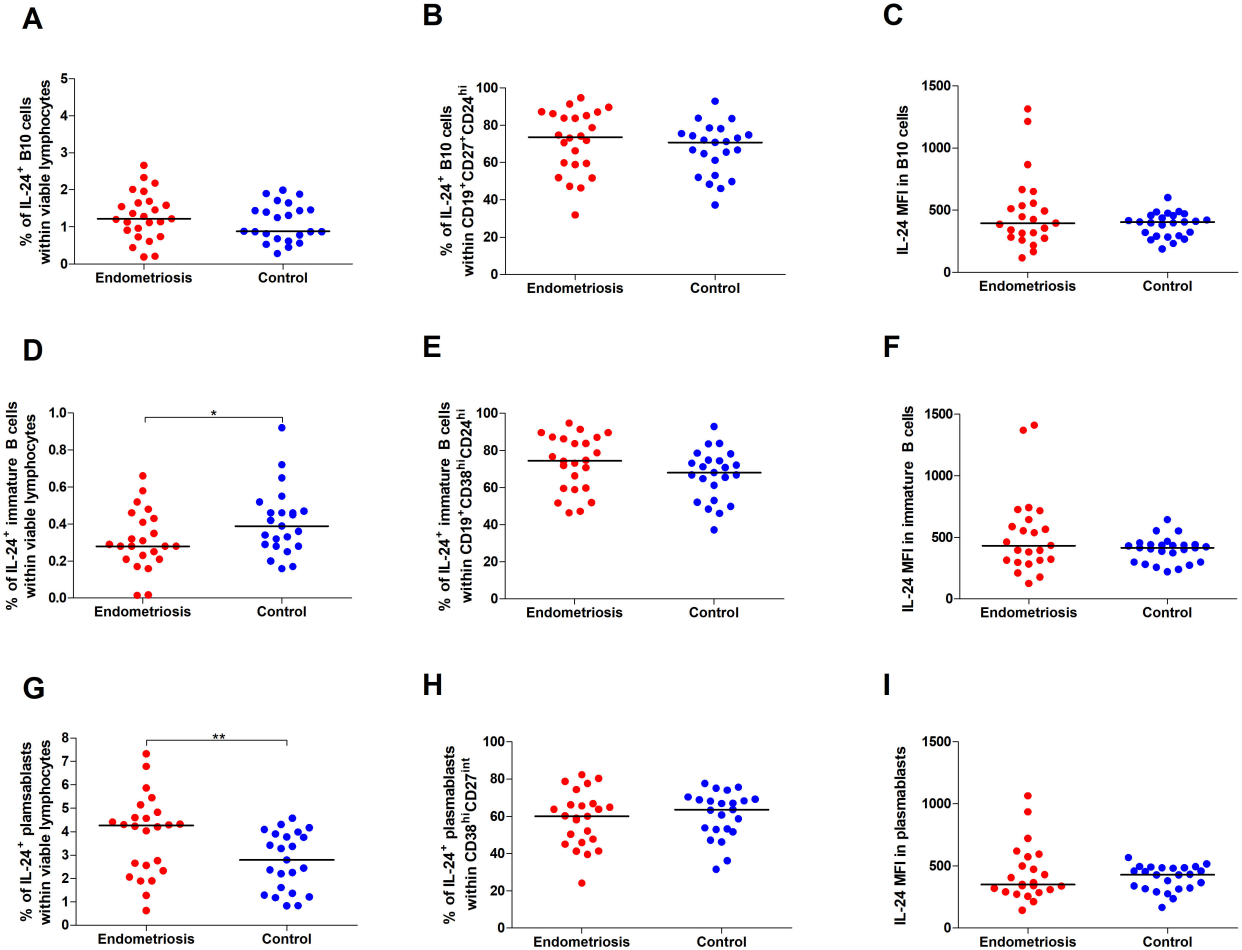
Percentage and IL-24 expression in selected subpopulations of Bregs in women with endometriosis compared with control. Whole peripheral blood obtained from women with endometriosis (red dots) and control group (blue dots) were stimulated with 12-myristate 13-acetate (PMA) and ionomycin in the presence of Brefeldin A and Monensin A. The following parameters were measured by flow cytometry: **(A)** Percentage of IL-24–producing B10 cells (CD19^+^CD27^+^CD24^hi^) within viable lymphocytes, **(B)** Percentage of IL-24-producing cells among B10 cells, **(C)** Median fluorescence intensity (MFI) of IL-24 within B10 cells, **(D)** frequency of IL-24–producing immature B cells (CD19^+^CD38^hi^CD24^hi^) within viable lymphocytes, **(E)** Percentage of IL-24-producing cells among immature B cells, **(F)** MFI of IL-24 within immature B cells, **(G)** Percentage of IL-24–producing plasmablasts (CD19^-^CD38^hi^CD27^int^) within viable lymphocytes, **(H)** Percentage of IL-24-producing cells among plasmablasts, and **(I)** MFI of IL-24 within plasmablasts. Data are presented as individual values with median. Data were analyzed using Student’s *t*-test (normal distribution) or Mann–Whitney *U* test (non-normal distribution). *p < 0.05 and **p < 0.01.

Comparing women with endometriosis with healthy women revealed no differences in the percentages of IL-24–producing B10 cells (**Figure 3A**) and IL-24–producing B10 cells among CD19^+^CD27^+^CD24^hi^ cells (**Figure 3B**), or median fluorescence intensity (MFI) of IL-24 in B10 cells (**Figure 3C**) (all p > 0.05).

**Figure 4:**
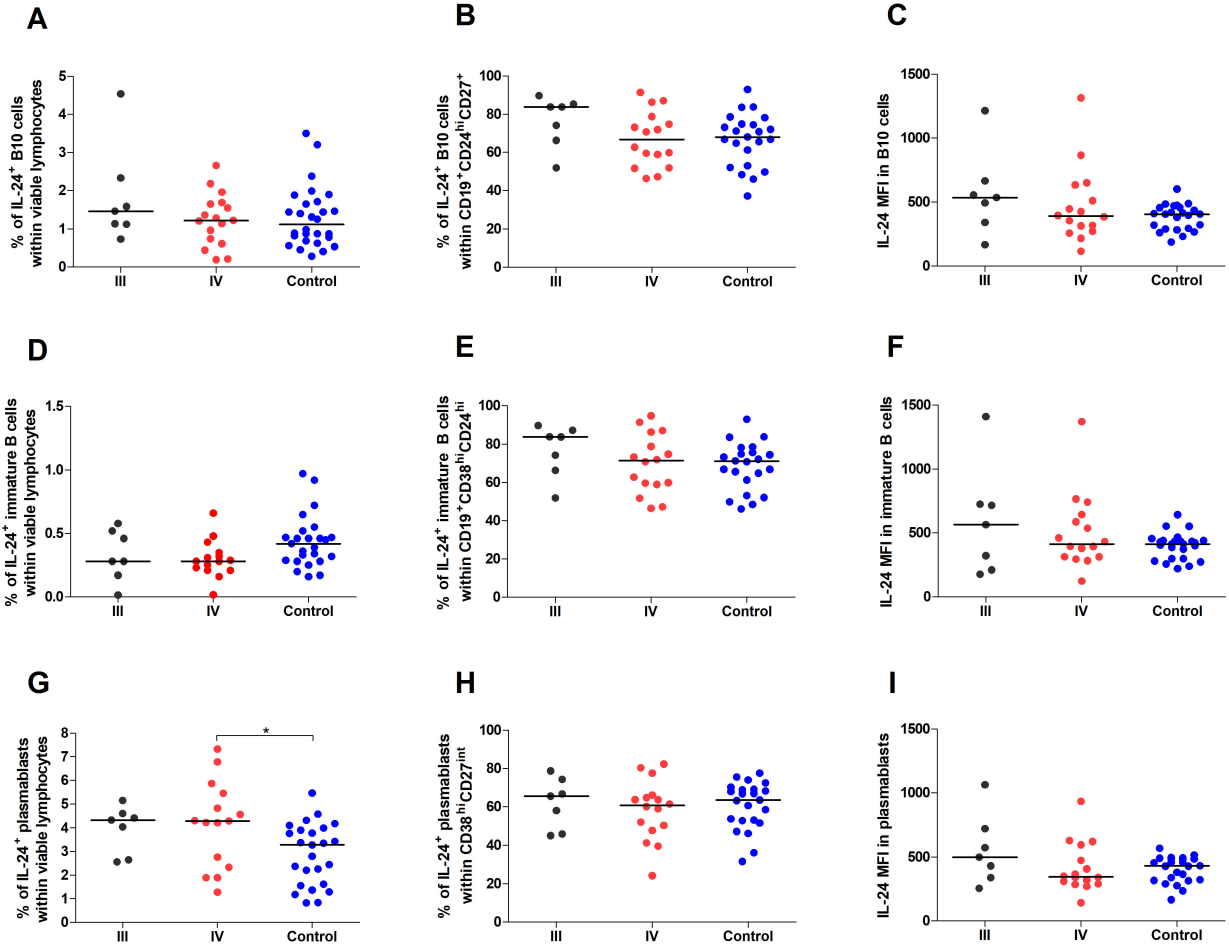
Percentage and IL-24 expression in Breg subpopulations stratified by endometriosis stage (III and IV) compared to control. Whole peripheral blood obtained from women with stages III (black dots) and IV (red dots) of endometriosis and control group (blue dots) were stimulated with PMA and ionomycin in the presence of Brefeldin A and Monensin A. The following parameters were measured by flow cytometry: **(A)** Percentage of IL-24–producing B10 cells (CD19^+^CD27^+^CD24^hi^) within viable lymphocytes, **(B)** Percentage of IL-24-producing cells among B10 cells, **(C)** Median fluorescence intensity (MFI) of IL-24 within B10 cells, **(D)** Percentage of IL-24–producing immature B cells (CD19^+^CD38^hi^CD24^hi^) within viable lymphocytes, **(E)** Percentage of IL-24-producing cells among immature B cells, **(F)** MFI of IL-24 within immature B cells, **(G)** Percentage of IL-24–producing plasmablasts (CD19^-^CD38^hi^CD27^int^) within viable lymphocytes, **(H)** Percentage of IL-24-producing cells among plasmablasts, and **(I)** MFI of IL-24 within plasmablasts. Data are presented as individual values with median. Statistical analysis was performed using one-way analysis of variance (ANOVA; normal distribution) or Kruskal–Wallis test (non-normal distribution) Dunnets’s multiple comparison *post hoc* test (p < 0.05). *p < 0.05.

However, we observed decreased percentages of IL-24–producing immature B cells in women with endometriosis compared with those in the control group (p < 0.05; **Figure 3D**). The percentage of IL-24–producing immature B cells among CD19^+^CD38^hi^CD24^hi^ cells (**Figure 3E**) and IL-24 (MFI) in these cells (**Figure 3F**) showed no significant differences between the two groups. Furthermore, the percentage of IL-24–producing plasmablasts was significantly higher in women with endometriosis (p < 0.01; **Figure 3G**), although the percentage of IL-24–producing plasmablasts among CD19^-^CD38^hi^CD27^int^ cells (**Figure 3H**) or their IL-24 expression levels (MFI; **Figure 3I**) was similar in both groups. No significant differences were observed in the percentages of IL-24–producing B10 cells (**Figure 4A**) and IL-24 producing B10 cells among CD19^+^CD27^+^CD24^hi^ cells (**Figure 4B**), as well as IL-24 expression in these cells (**Figure 4C**) between healthy and endometriosis-positive groups (all p > 0.05). Similarly, immature B cells among CD19^+^CD38^hi^CD24^hi^ cells showed no significant difference) between control, stage III, and stage IV groups (**Figures 4D–F**; all p > 0.05). However, the percentages of IL-24–producing plasmablasts (**Figure 4G**) were significantly higher in women with stage IV endometriosis (p < 0.05) compared with those in the control group. No differences were observed in the percentage of IL-24–producing plasmablasts among CD19^-^CD38^hi^CD27^int^ cells (**Figure 4H**) and IL-24 expression in these cells (**Figure 4I**; p > 0.05).

### 3.2 Women with endometriosis exhibit higher percentage of IL-24–producing Tregs

**Figure 5:**
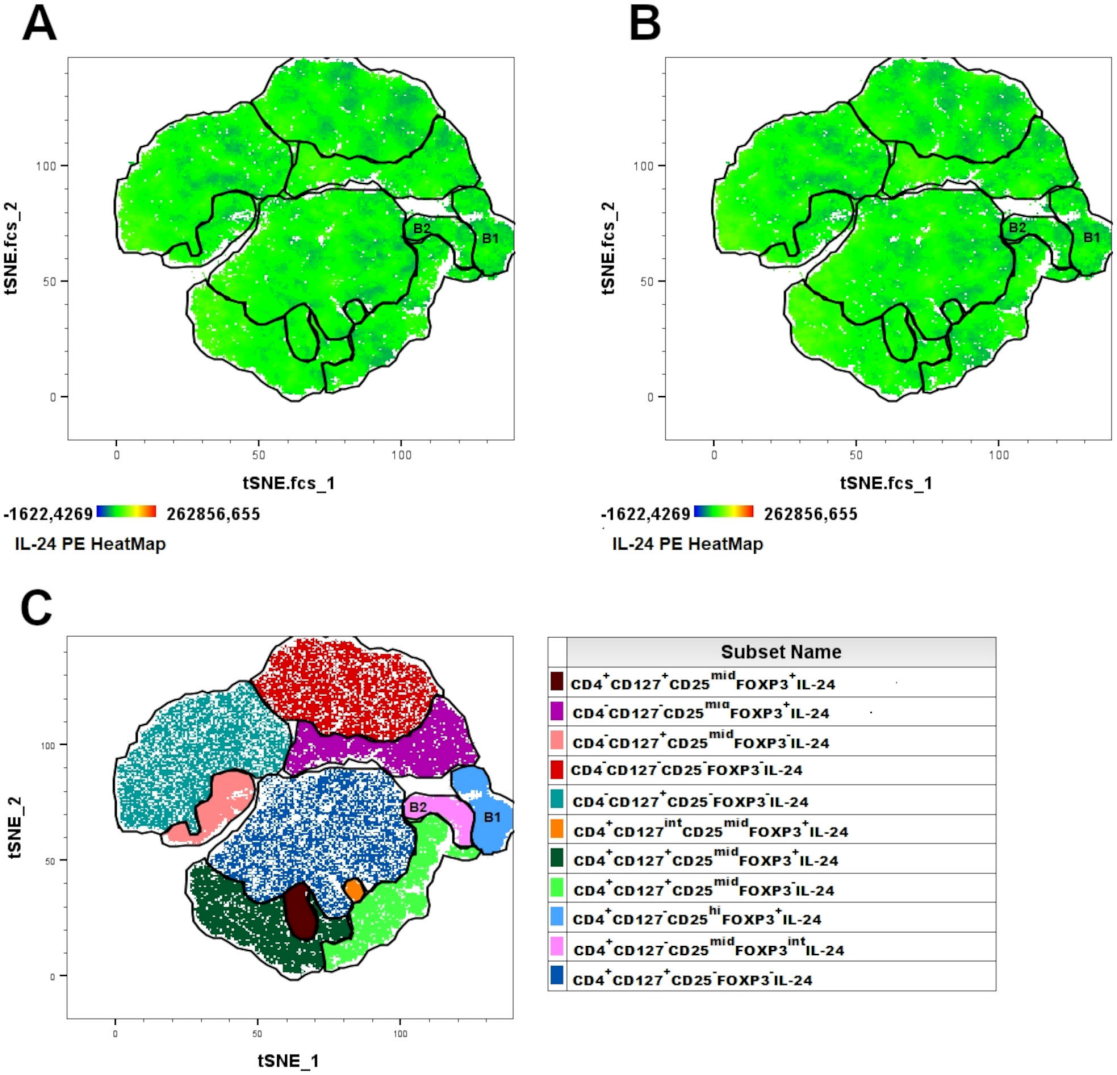
Combined t-SNE heatmap of IL-24–producing T cells. Combined t-SNE analysis was performed using 24 paired samples obtained from healthy women **(A)** and women with endometriosis **(B)**. **(C)** t-SNE plot presenting 11 distinct clusters of IL-24–producing cells identified using FLOWSOM algorithm. Regulatory T lymphocytes were identified in B1 and B2 clusters.

We explored whether Tregs produce IL-24 and whether their percentages change in women with endometriosis. Using the tSNE algorithm, we visualized IL-24–producing T cells in healthy controls (**Figure 5A**) and women with endometriosis (**Figure 5B**). Next, the FLOWSOM algorithm was used to identify distinct clusters of IL-24–producing cells (**Figure 5C**). IL-24–producing T cell heatmap revealed the following Tregs clusters: B1 (CD4^+^CD127^-^CD25^hi^FOXP3^+^IL-24^+^) and B2 (CD4^+^CD127^-^CD25^mid^FOXP3^int^ IL-24^+^). tSNE analysis, presented as IL-24 heatmaps in T cell subsets, showed that all the analyzed clusters expressed IL-24 and that no significant differences were observed in the complexity of T cell clusters or distribution of IL-24 between women suffering from endometriosis (**Figure 5A**) and healthy controls (**Figure 5B**).

**Figure 6:**
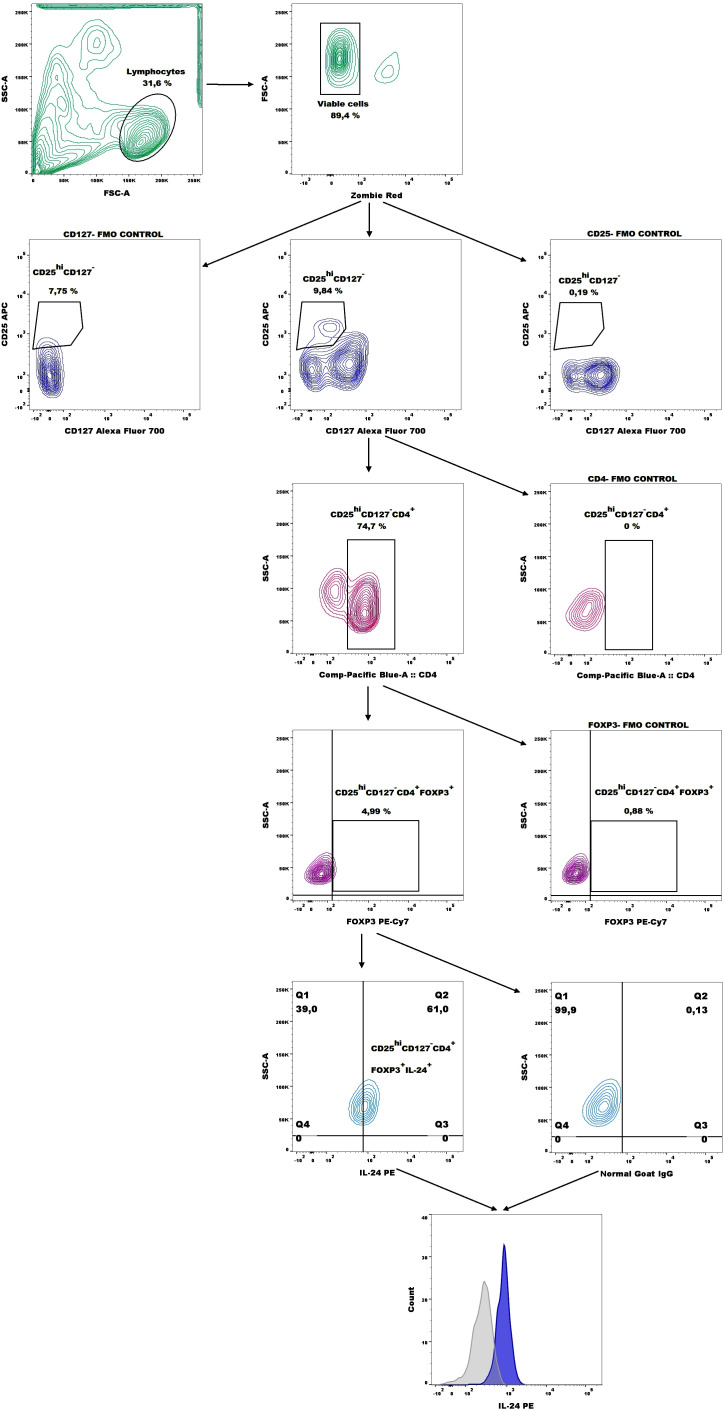
Representative dot plots showing the gating strategy for IL-24–producing Tregs (CD4^+^CD127^-^CD25^hi^FOXP3^+^ cells) and representative histograms of IL-24 expression (blue histogram) overlaid with the respective isotype-matched control (grey histogram) derived from whole blood of patients with endometriosis.

**Figure 7:**
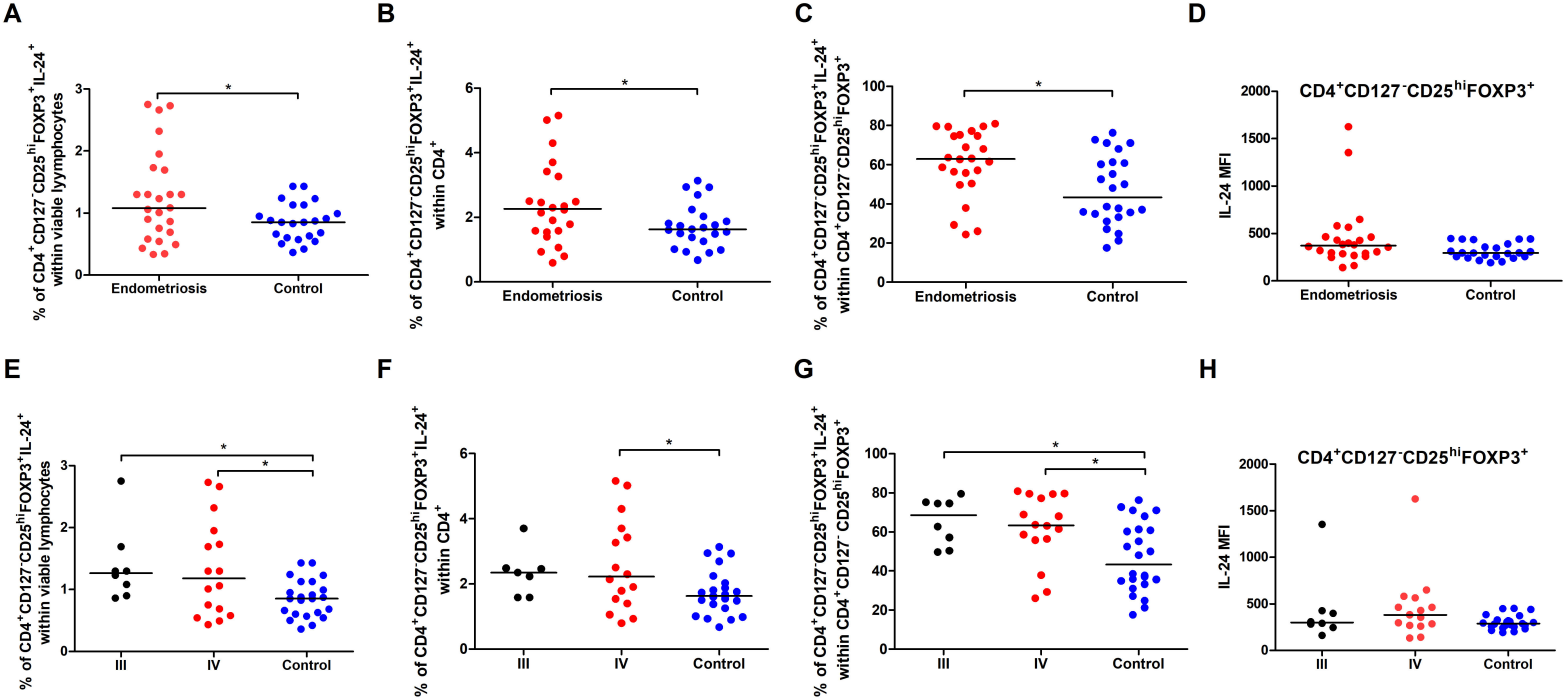
Percentage and IL-24 expression in Tregs in total pool of women with endometriosis (red dots in **A–D**) and women with stages III (black dots in **E–H**) and IV (red dots in **E–H**) endometriosis compared to control (blue dots). Whole peripheral blood were stimulated with PMA and ionomycin in the presence of Brefeldin A and Monensin A. The following parameters were measured by flow cytometry: (**A, E)** Percentage of IL-24–producing Tregs (CD4^+^CD127^-^CD25^hi^FOXP3^+^) within viable lymphocytes, **(B, F)** Percentage of IL-24–producing Tregs within CD4^+^ cells, **(C, G)** Percentage of IL-24–producing Tregs among total Tregs, and **(D, H)** Median fluorescence intensity (MFI) of IL-24 within Tregs. Data are presented as individual values with median. Data presented in graphs a-d were analyzed using Student’s *t*-test (normal distribution) or Mann–Whitney *U* test (non-normal distribution), whereas data in graphs e-f were analyzed using one-way ANOVA (normal distribution) or Kruskal–Wallis test (non-normal distribution) with Dunnets’s multiple comparison *post-hoc* test (p < 0.05). *p < 0.05.

Next, we analyzed the percentage of CD4^+^CD127^-^CD25^hi^FOXP3^+^IL-24^+^ (IL-24–producing Tregs) based on the gating strategy shown in **Figure 6**. Women with endometriosis showed a higher percentage of IL-24–producing Tregs (CD4^+^CD127^-^CD25^hi^FOXP3^+^IL-24^+^) (**Figure 7A**), IL-24–producing Tregs among CD4^+^ cells (**Figure 7B**), and among Tregs (**Figure 7C**) in comparison to controls (both p < 0.05). However, no differences were observed in IL-24 expression (IL-24 MFI) in Tregs among the examined groups (p > 0.05; **Figure 7D**). Similar results were noted when the data were stratified by the American Society for Reproductive Medicine (ASRM) classification of endometriosis (**Figures 7E, G, H**). The only exception was that the percentage of CD4^+^CD127^-^CD25^hi^FOXP3^+^ IL-24^+^ cells within CD4^+^ cells did not differ between women with stage III endometriosis compared with that in the control group (**Figure 7F**).

## 4 Discussion

In endometriosis, the growth of endometrial tissue outside the uterine cavity disrupts peripheral and local tissue homeostasis. The dysfunctional response of peripheral and peritoneal cavity immune cells has been associated with the development of endometriosis (20, 21, 49, 50). Regulatory cells play a fundamental role in developing suppressive immune responses that allow endometriotic fragments to grow or spread outside the uterine cavity. Among these, Tregs and Bregs are key players in establishing self-tolerance. Their regulatory abilities are linked to the secretion of inhibitory cytokines, such as IL-35, TGF-beta, and IL-10, all of which are dysregulated in endometriosis. IL-24, a member of the IL-10 cytokine family, has also been implicated in the pathogenesis of endometriosis (33).

IL-24 plays a significant role in immune responses, particularly in cancer. As a tumor-suppressive cytokine, IL-24 induces apoptosis in cancer cells, while sparing normal cells, and inhibiting angiogenesis within the tumor, thereby restricting tumor growth and spread and enhancing the immune system’s ability to detect and destroy tumor cells (34, 35, 37–40, 51). IL-24 is produced by both non-immune cells like astrocytes, keratinocytes, pancreatic myofibroblasts, and endothelial cells (52–54) and immune cell populations such as monocytes, macrophages, dendritic cells, NK cells, T cells, and B cells (42–45). It is inducible in peripheral blood mononuclear cells (PBMCs) by pro-inflammatory cytokines, suggesting a systemic immunomodulatory role (45). Macrophages and NK cells produce IL-24 under the control of STAT3 and STAT4, respectively, reflecting their distinct roles in inflammation (42, 43). Furthermore, activated T helper (Th) cells stimulate macrophages to produce IL-24, leading to the suppression of mammary tumor growth (55). In B cells, IL-24 acts as a negative regulator of humoral immunity by inhibiting plasma cell differentiation (44). In non-immune contexts, IL-24 contributes to epithelial inflammation in the skin (52), modulates fibrogenic activity in pancreatic myofibroblasts (53), and exerts cytoprotective effects in endothelial cells under oxidative stress conditions (54). Taken together, the aforementioned studies indicate the multifunctional nature of IL-24 in regulating a diverse range of cellular responses across both immune and non-immune cell types. IL-24 exhibits dose-dependent and context-sensitive biological activity, which underlies its paradoxical roles in inflammation, immune regulation, and cancer. At low concentrations, IL-24 acts predominantly as an immunomodulatory cytokine, particularly in T cells (56). Furthermore, low-dose IL-24 has been shown to promote immune homeostasis by inducing IL-10 production in T helper 17 (Th17) cells, modulating mitochondrial signaling, and reducing signal transducer and activator of transcription 3 (STAT3) activity, thereby limiting the production of excessive pro-inflammatory cytokines (57, 58). This regulatory role is especially evident in autoimmune conditions, where IL-24 helps limit excessive immune activation. At moderate concentrations, IL-24 signals through IL-10 family receptors (IL-20RA/IL-20RB and IL-22RA1/IL-20RB) on immune and epithelial cells, promoting the production of cytokines and tissue repair mediators, which supports immune activation and maintains epithelial barrier integrity (59). At high concentrations, IL-24 activates the proteins PERK and eIF2, leading to reduced expression of survival proteins and simultaneously activating a ceramide-dependent pathway that induces apoptosis in tumor cells (60). Notably, IL-24’s cytotoxicity in cancer cells can also occur independently of receptor engagement, further highlighting its selective action toward malignant cells (61). Furthermore, IL-24’s function is heavily influenced by the tissue microenvironment (34). For example, in cancer models, its anti-tumor effects can be both direct through local overexpression or therapeutic delivery and indirect, mediated by immune cells (62–65).

Dysregulation of IL-24 expression has been linked to several autoimmune diseases such as psoriasis (66), inflammatory bowel disease (67), and rheumatoid arthritis (19). The immune-related changes observed in endometriosis closely resemble those found in autoimmune diseases, suggesting a shared underlying pathogenesis between the two conditions. Individuals with endometriosis show signs of systemic immune dysregulation and face an increased risk, ranging from 30% to 80% of developing immune-related comorbidities, including classical autoimmune diseases such as rheumatoid arthritis, multiple sclerosis, and coeliac disease; autoinflammatory conditions like osteoarthritis and mixed-pattern disorders such as psoriasis (68–70). Additionally, as reported by Shigesi et al. (2025), endometriosis shares a significant genetic correlation with both rheumatoid arthritis and multiple sclerosis. The presence of concurrent autoimmune diseases has also been associated with a more severe progression of endometriosis (71). As noted previously, interleukin-24 (IL-24) can activate various immune cells, including macrophages, dendritic cells, and T lymphocytes, leading to increased production of pro-inflammatory cytokines and reactive oxygen species. This immune activation contributes to the maintenance of chronic inflammation and progressive tissue damage. In the context of endometriosis, IL-24–driven inflammatory signaling may intensify local immune activation, contribute to the persistence of ectopic endometrial lesions, and disrupt the structural integrity of surrounding tissues, including the peritoneum and reproductive organs. The only study that addressed IL-24 expression in endometriosis was by Shao et al. (2016), which reported reduced IL-24 expression in the eutopic and ectopic endometrium of women with endometriosis compared with women without the condition. They demonstrated that monocyte-derived macrophages from healthy women and co-cultured with the eutopic endometrium of women with endometriosis downregulated IL-24 production and its receptor expression (calculated as MFI) compared to endometrial stromal cells cultured alone. Moreover, they revealed that macrophages significantly restricted the inhibitory effects of IL-24 on the viability, invasion, and proliferation markers of endometrial stromal cells. Although these findings were interesting, the experimental conditions and selection of control group were not in line with physiological conditions. Specifically, macrophages from healthy women were co-cultured with antigenically foreign endometria, in which created an artificial system conducive to immune responses against foreign endometrium. A more physiologically relevant approach would involve immune cells from women with endometriosis that would reflect the altered cytokine milieu characteristic of the disease (15–24).

In our study, we aimed to assess whether regulatory cells in their most “natural” state produce IL-24 and whether endometriosis is characterized by altered IL-24 expression. Unlike Shao et al., 2016 (33), who investigated local expression, we examined IL-24 in peripheral blood. These differences in scientific questions and experimental design likely account for observed discrepancies between our findings and their results. We revealed for the first time that circulating regulatory T and B lymphocytes secrete IL-24 and their percentages are altered in women with endometriosis compared with healthy women. Other studies have mainly investigated IL-24 expression at the mRNA level, but rarely at the protein level (western blotting, ELISA, and immunochemistry), and predominantly in non-immune or basic immune cells (activated T cells, NK cells, or B cells) (41–45, 53, 56, 72). Our results, based on flow cytometry, indicated that over 60% of Tregs and Bregs secrete IL-24. However, the relative IL-24 expression in these cells (as measured by MFI) was similar in both groups. Nonetheless, we observed an increase in IL-24^+^ Tregs percentage in women with endometriosis, regardless of endometriosis stages. This suggests that the functional capacity of individual IL-24^+^ Tregs cells remains relatively stable, and the observed changes are primarily due to variations in the number of IL-24^+^ cells rather than altered expression per cell. Previously, Tregs percentage in women with endometriosis was reported to be higher than in healthy women (25, 26, 28), with proportions increasing with the disease progresses (33). The influence of Tregs on endometriosis may be multifaceted, as they are responsible for shaping the immune environment by modulating the behavior of other immune cells. For example in ovarian ectopic lesions, Tregs contribute to macrophage polarization toward the anti-inflammatory M2 phenotype (73). Furthermore, Tregs can also influence the activity of T helper cells, cytotoxic CD8^+^ T cells, and NK cells, either by suppressing or promoting their inflammatory responses (74). This indicates that Tregs may, both directly and indirectly, contribute to the pathogenesis of endometriosis by promoting the adhesion and growth of endometrial implants. However, the exact role of IL-24^+^ Tregs in immunotolerance remains unclear. IL-24 may directly influence Tregs differentiation and function by modulating cytokine environments, or indirectly affect the balance between pro-inflammatory and anti-inflammatory responses, affecting Tregs activity in immune regulation (75). Zhang et al., 2019 (56) showed that low IL-24 concentration promotes *FoxP3* mRNA expression in tumor-infiltrating T cells, whereas high concentrations decrease Tregs percentage, *FoxP3* mRNA expression, and IL-10/IL-35 secretion in cells obtained from patients with colorectal adenocarcinoma. The only other study describing the influence of IL-24 on Tregs showed that this cytokine plays a crucial role in enhancing the suppressive function of mice CD4^+^CD25^+^ T cells/Peyer’s patch B cells co-cultures, named Treg-of-B (P) cells (76). In the absence of IL-24, the Treg-of-B (P) cells exhibited a decreased ability to inhibit responder T cell proliferation, whereas IL-24 blocking impaired the suppressive function of Treg-of-B (P) cells. They further suggested that IL-24 functions through autocrine signaling to stimulate IL-10 expression in Treg-of-B (P) cells. Therefore, it is tempting to speculate the induction in IL-24^+^ Tregs observed in our study might have enhanced adverse immune tolerance in endometriosis.

In the present study, we also determined the percentages of IL-24–producing Bregs. Bregs may help regulate the inflammatory milieu in endometriosis. They suppress autoreactive T cell activation, reduce pro-inflammatory cytokine production, and promote tissue repair (77–81). This function may be compromised in individuals with endometriosis, which could contribute to persistent inflammation and disease development. In our previous study, we demonstrated that B10 cell and plasmablast percentages were lower in women with endometriosis (32). Simultaneously, we revealed that the percentages of the same Breg subpopulation expressing IL-35 were higher in patients with endometriosis. Here, we also observed a similar trend of increased IL-24^+^ plasmablast percentages in women with endometriosis compared with the healthy controls. Plasmablasts are B cells that might secrete antibodies that contribute to tissue damage and inflammation in the pelvic region, which causes pain and fibrosis characteristic of endometriosis. Patients with endometriosis exhibit an increased prevalence of anti-endometrial (82) as well as IgG and IgM autoantibodies that target phospholipids, histones, or DNA (83). Another study demonstrated the significant presence of plasma cells and macrophages in endometriotic lesions (15). Normally, B cells mature into plasma cells that produce antibodies. However, in endometriosis, plasmablasts may be inappropriately activated, leading to an overproduction of antibodies that attack the body’s own tissues. The role of IL-24 in B cell function is still being actively studied, especially in cancer, with research suggesting that it may influence both B cell activation and regulation. IL-24 is expressed in naïve and memory B cells but is repressed in centroblasts (44). Moreover, the same authors reported that CD27^+^ memory B cells and CD5^+^ B cells exhibit elevated IL-24 expression upon BCR activation and CD40–CD40L ligation. *In vitro* experiments have demonstrated that IL-24 promotes CD40L-induced B cell proliferation, but inhibits plasma cell formation, immunoglobulin (Ig) G production, and IL-10 expression (44). Conversely, another study found that IL-24 induces B cell apoptosis by activating genes involved in the mitochondrial apoptotic pathway during the later stages of B cell differentiation, while inhibiting genes related to DNA replication and metabolism in the early stages (84). The aforementioned findings provide valuable insights, yet the precise impact of IL-24 on B cell differentiation and function, including plasmablasts, remains unclear. A deeper understanding of how this cytokine influences B cell activation, antibody production, and immune regulation in disease contexts could open up new potential treatment strategies.

Despite the novel findings on the role of IL-24 in endometriosis, our study has several limitations that should be considered when interpreting the results. Firstly, our study lacks patients with early stages (stage I/II) of endometriosis, which is due to diagnostic challenges as the disease is often asymptomatic or undiagnosed until it reaches more advanced stages. This limits the ability to assess the dynamics of interleukin-24 in the full spectrum of disease progression. Secondly, the study’s focus on peripheral blood samples excludes the analysis of peritoneal fluid or ectopic endometrial lesions, which could offer additional insights into the local immune microenvironment where IL-24 may play a role. Thirdly, stimulation of cells with PMA and ionomycin, may non-specifically induce IL-24 expression, potentially confounding the interpretation of cytokine levels in the context of physiological conditions. However, the PMA/Ionomycin stimulation is a well-established approach for inducing cytokine production. In our study, the stimulation conditions were consistent across both groups, thereby eliminating the potential confounding effect of the stimulation procedure. Finally, the inclusion of functional assays in upcoming research may help elucidate whether IL-24 secretion is associated with the immunosuppressive activity of regulatory cells. Addressing these limitations in future studies will be essential for a deeper understanding of IL-24’s role in the progression of endometriosis, and its potential clinical relevance.

In conclusion, we have shown for the first time that circulating regulatory T cells and selected B cell subpopulations (plasmablasts, immature B cells and B10 cells) secrete IL-24. Interestingly, their percentages are altered in women with endometriosis compared to that in healthy women. Based on our findings, we speculate that IL-24–producing cells with a regulatory phenotype are involved in the pathogenesis of endometriosis. IL-24 secretion appears to enhance the improper immunosuppressive activity of Tregs and plasmablasts in endometriosis, which enables the implantation and growth of endometrial lesions outside the uterus. Further research is required to elucidate the exact role of IL-24–producing regulatory lymphocytes in the development and progression of endometriosis.

## Data Availability

The raw data supporting the conclusions of this article will be made available by the authors, without undue reservation.
